# A mass spectrometry-based proteomics strategy to detect long-chain *S*-acylated peptides

**DOI:** 10.1039/d5an00557d

**Published:** 2025-08-28

**Authors:** Samiksha Sardana, Andrea Trezza, Francine Rodrigues Ianiski, Anneroos E. Nederstigt, Marc P. Baggelaar

**Affiliations:** a Biomolecular Mass Spectrometry and Proteomics, Bijvoet Center for Biomolecular Research and Utrecht Institute for Pharmaceutical Sciences, University of Utrecht Padualaan 8 Utrecht 3584 CH The Netherlands m.p.baggelaar@uu.nl; b Netherlands Proteomics Center Padualaan 8 Utrecht 3584 CH The Netherlands

## Abstract

Long-chain *S*-acylation is the addition of long-chain fatty acids to cysteine residues on proteins. This lipid modification is essential for protein membrane association and signalling but presents analytical challenges due to both its hydrophobicity and the labile nature of thioester bonds. We developed and optimised a bottom-up mass spectrometry workflow tailored for the detection of long-chain *S*-acylated peptides. Following liquid chromatography optimisation for improved separation and elution of long-chain *S*-acylated peptides from a C_18_ stationary phase, we investigated thioester stability under typical proteomics sample preparation conditions, including variations in pH, reducing agents, and trypsin digestion. Stability analyses revealed that long-chain *S*-acylated peptides were generally resistant to pH variations and reducing agents, while extended digestion times resulted in a loss of signal from some peptides. For MS/MS analysis, CID, HCD and ETD were applied to analyse long-chain *S*-acylated peptides. Neutral losses of the modification were observed with all these fragmentation methods. However, HCD proved to be the most effective, as the fragment ions resulting from the neutral losses provided sequence information, unlike those from CID and ETD. Applying this workflow to HEK293T cells overexpressing the long-chain *S*-acylated proteins GNA13 and RhoB, we detected dual acylation states of GNA13 and observed both long-chain *S*-acylation and prenylation on RhoB. Our optimised analytical strategy facilitates the identification and analysis of long-chain *S*-acylation on proteins without the need for chemical derivatization by alkyne-tagged probes or acyl-biotin exchange. Although recombinant overexpression of the long-chain *S*-acylated proteins was still required for long-chain *S*-acylation detection, this direct analysis strategy for protein long-chain *S*-acylation enables the study of lipid modifications with lipid-specific resolution, laying a foundation for deeper insights into the regulatory roles of these hydrophobic modifications in protein function and cellular signalling.

## Abstract 2

Systematic evaluation of each step in the bottom-up proteomics workflow for its compatibility with long-chain *S*-acylated peptide detection.

## Graphical abstract

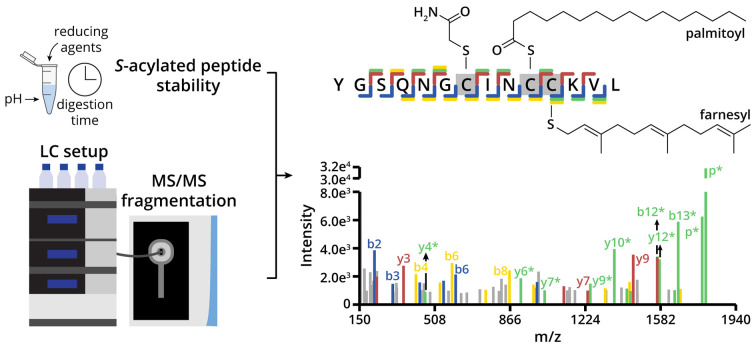

## Introduction

Protein long-chain *S*-acylation refers to the covalent attachment of C_14_–C_20_ fatty acids to cysteine residues, which enhances protein hydrophobicity and membrane affinity.^1,2^ Often referred to as *S*-palmitoylation due to the frequent attachment of C16:0, this modification plays a crucial role in regulating protein subcellular localisation, protein–protein interactions, and intracellular signalling pathways.^3–5^ It is estimated that over 3000 proteins in the human proteome undergo long-chain *S*-acylation, highlighting its critical role in both health and disease.^5–11^ In contrast to other lipid modifications such as *N*-myristoylation, *N*-acylation, and *O*-acylation, which target different amino acids and involve various fatty acid chains,^12^*S*-acylation stands out for its reversible nature and dynamic regulation. Long-chain *S*-acylation is catalysed by a family of 23 enzymes known as protein acyltransferases (PATs), which share a highly conserved Asp-His-His-Cys (DHHC) tetrapeptide motif in their active sites.^13^ The removal of fatty acids is mainly achieved by thioesterases, including acyl-protein thioesterase 1 and 2 (APT1/2), palmitoyl-protein thioesterase 1 (PPT1) and the α/β hydrolase domain-containing protein 17 (ABHD17) family.^10,14^ Dysregulation of this dynamic post-translational modification (PTM) has been implicated in a wide range of diseases, including cancer, immune disorders, and neurodegenerative diseases.^15–19^ Therefore, a comprehensive understanding of the extent and regulation of long-chain *S*-acylation could lead to novel therapeutic interventions to target these diseases.

Despite its prevalence and biological significance, our understanding of long-chain *S*-acylation remains limited compared to other common PTMs such as phosphorylation, acetylation, and glycosylation.^20–22^ This knowledge gap largely arises from challenges in detecting long-chain *S*-acylated proteins due to low stoichiometric levels, hydrophobicity, and the instability of the thioester bond. To circumvent these challenges, indirect methods that combine chemical biology strategies with bottom-up mass spectrometry have been developed to monitor long-chain *S*-acylation. Two main approaches – acyl-biotin exchange (ABE) and lipid metabolic labelling (LML) – have been effectively used to identify long-chain *S*-acylated proteins.^23–28^ ABE, a cysteine-centric approach, enriches long-chain *S*-acylated proteins by tagging them with biotin after nucleophilic cleavage of thioester bonds by hydroxylamine, enabling large-scale analysis by bottom-up proteomics. The second method, LML, uses fatty acid analogues like 15-hexadecynoic acid to modify long-chain *S*-acylated proteins, followed by biotin tagging through click chemistry.^29^

While these indirect methods have significantly advanced our understanding of long-chain *S*-acylation in various cellular processes, they have inherent limitations. ABE involves extensive blocking of free cysteines and nucleophilic cleavage of the thioester bond, which results in the loss of lipid information and may increase the risk of false-positive identifications when capping is incomplete. The LML strategy relies on exogenous fatty acids, is not compatible with the analysis of long-chain *S*-acylation in tissues, may alter the metabolic state of the cells, and cannot provide information about endogenous lipids attached to proteins. Direct detection of long-chain *S*-acylation can overcome these issues; thus, a methodology that enables efficient detection of endogenous long-chain *S*-acylation is highly desired. Several efforts in that direction have been attempted before.^28,30–36^ However, a robust strategy for the direct detection of long-chain *S*-acylation is still lacking. To address this challenge, we systematically evaluated each step in the bottom-up proteomics workflow for its compatibility with long-chain *S*-acylated peptide detection. Emphasis was put on the key features of long-chain *S*-acylation that impede detection of this modification in standard bottom-up proteomics workflows, namely, hydrophobicity and thioester bond lability.

First, we optimised the retention and elution of long-chain *S*-acylated peptides in reversed-phase liquid chromatography (RP-LC) by using synthetic long-chain *S*-acylated peptides. Second, we explored different MS/MS fragmentation methods to gain an understanding of the fragmentation behaviour of long-chain *S*-acylated peptides. Third, we examined the stability of the thioester bond of synthetic long-chain *S*-acylated peptides during various steps common in bottom-up proteomics workflows. Finally, the identified optimal parameters for long-chain *S*-acylated peptide detection were applied to detect lipidated peptides in full proteomes from HEK293T cells that recombinantly expressed GNA13 and RhoB.

## Materials and methods

### *S*-Palmitoylation of synthetic peptides

*S*-Palmitoylated peptides were synthesized following a previously published protocol.^37^ Each synthetic peptide (250 μg, 152–286 nmol, Genscript) was dissolved in 12.5 μL of trifluoroacetic acid (TFA). Then, 1.25 μL of palmitoyl chloride (4.12 μmol, Sigma-Aldrich) was added, and the reaction mixture was incubated for 10 min at RT with continuous shaking. After the reaction, 240 μL of methanol and 1 mL of Milli-Q water (MQ, Millipore) were added. Solvents were removed by lyophilisation (FreeZone Freeze Dryer, Labconco). The dried peptides were dissolved in a 1 : 1 (v/v) mixture of acetonitrile and methanol, combined (peptide 1/peptide 2/peptide 3/peptide 4 = >1.28 : 1.28 : 1.00 : 1.13 in moles), aliquoted, and lyophilised until LC-MS/MS analysis.

### Cell culture

HEK293T (CRL-11268, ATCC) with a passage number below 20 was cultured in Dulbecco's modified Eagle's medium (Capricorn Scientific) supplemented with l-glutamine, sodium pyruvate, 10% foetal bovine serum (Gibco), and penicillin/streptomycin (100 U mL^−1^, 100 μg mL^−1^ Gibco). Cells were maintained under a humidified atmosphere at 37 °C with 5% CO_2_. For harvesting, cells were cultured in T175 flasks until confluency, washed twice with cold Dulbecco's phosphate-buffered saline (without Ca & Mg, Capricorn Scientific), and flash frozen. Cell pellets were stored at −80 °C until use.

### Cell lysis and enzymatic digestion

Frozen HEK293T cell pellets were lysed in 50 mM Tris-HCl pH 7.5, 150 mM NaCl, 5 mM EDTA pH 7.5, 4% (w/v) SDS, 500 nM Palmostatin B (Sigma-Aldrich), 1× complete EDTA-free protease inhibitor cocktail (Roche), and 1 mM PMSF. The cells were sonicated at 70% amplitude with 0.5 s cycles (Hielscher Ultrasonics). Following sonication, the lysates were centrifuged at 16 000*g* for 10 min, and the supernatant was collected. Proteins were precipitated by methanol/chloroform precipitation (4 volumes of methanol, 1 volume of chloroform, and 3 volumes of MQ). The resulting protein pellets were resuspended in lysis buffer, and the protein concentration was measured using a bicinchoninic acid protein assay (BCA assay, Thermo Fisher Scientific).

For reduction and alkylation, TCEP (10 mM final concentration) was added to HEK293T lysates (2 mg, 3 mg mL^−1^), and the samples were incubated for 1 h at RT, followed by *N*-ethylmaleimide (NEM) addition (final concentration 50 mM) for another hour at RT. Proteins were precipitated, resuspended in 1 mL of 50 mM Tris-HCl pH 7.5, and digested with trypsin (1 : 50 w/w, Sigma-Aldrich) o/n at 37 °C. Digestion was quenched to a final concentration of 0.1% TFA, and peptides were desalted using an Oasis HLB μElution plate (Waters) with sequential elution: (1) 70% acetonitrile with 0.1% TFA and (2) 85% acetonitrile, 15% isopropanol with 0.1% TFA. The peptide solution was dried by lyophilisation and stored at −20 °C until spike experiments.

### Assessment of thioester stability

To assess stability at various pH levels, HEK293T digests (20 μg) were dissolved in 20 μL of 50 mM HEPES (pH 6.5, 7.0, 7.5 or 8.0) and spiked with either 1 μL of *S*-palmitoylated peptide solution (1 μg, in methanol) or 1 μL of methanol (control). After 2 hour of incubation at RT, 0.45 μL of TFA was added to all samples. The *S*-palmitoylated peptide-spiked samples were treated with 1 μL of methanol, while the control (methanol) samples received 1 μL of *S*-palmitoylated peptide solution. Samples were diluted to 100 μL with 50 mM HEPES pH 7.5, acidified with TFA to a final concentration of 1%, desalted, and lyophilised as described above.

For stability experiments with hydroxylamine (HA), reducing agents and digestion conditions, HEK293T digests (20 μg) were dissolved in 20 μL of 50 mM HEPES pH 7.5, and spiked with either 1 μL of *S*-palmitoylated peptide solution (1 μg) or 1 μL of methanol (control). To assess stability in the presence of HA, the samples were incubated with 1 M HA for 1 h at RT. For stability testing with reducing agents, the samples were treated with either TCEP or DTT to a final concentration of 10 mM for 1 h at RT. To assess stability during digestion, the samples were incubated with trypsin (0.4 ng) for either 8 h or 16 h at 37 °C. In all three cases, after incubation, 0.45 μL of TFA was added to all the samples. Then, the *S*-palmitoylated peptide-spiked samples were treated with 1 μL of methanol, while the control (methanol) samples received 1 μL of *S*-palmitoylated peptide solution. Samples were diluted to 100 μL with 50 mM HEPES pH 7.5, acidified with TFA to a final concentration of 1%, desalted and lyophilised as described above.

### Transfection, lysis, and enzymatic digestion

HEK293T cells (3.5 × 10^6^ cells per 175 cm^2^) were plated in two T-175 flasks 24 h before transfection. Fresh full-growth medium (25 mL) was added before transfection with 18.2 μg of customised pcDNA3.1(+) vector (GenScript) of either wild-type GNA13 (including a C-terminal His_6_ tag and Strep-tag II, cloned in by KpnI/EcoRI) or wild-type RhoB (including an N-terminal Strep-tag II and His_6_ tag, cloned in by NheI/KpnI) using jetPRIME transfection reagent (polyplus) and jetPRIME buffer. After 48 h of incubation at 37 °C, cells were harvested (two flasks combined) and lysed in 50 mM HEPES pH 7.5, 150 mM NaCl, 1% (w/v) *n*-dodecyl-β-d-maltopyranoside (DDM, Santa Cruz Biotechnology), 500 nM Palmostatin B, and 1× complete EDTA-free protease inhibitor cocktail. Sonication (4× for 15 s at 70% amplitude) was used to aid solubilisation. The lysates were incubated with gentle stirring using a small magnetic bar for 2 h at 4 °C. The lysates were centrifuged (20 000*g* at 4 °C for 30 min), and the supernatant was collected. Supernatant (10 μL) and pellet (10 μL) samples were diluted with 50 mM HEPES pH 7.5 to reduce the DDM concentration to 0.1%. The pellet samples were sonicated. Both samples were reduced with TCEP (15 mM final concentration) for 15 min at RT and alkylated with chloroacetamide (20 mM) for 20 min at RT. The samples were digested with LysC (0.2 μg, Wako) at 37 °C for 1 h, followed by overnight trypsin (0.2 μg) digestion. Methanol (5% v/v) was added to each sample, which was then acidified with TFA (final concentration = 1% v/v). After centrifugation, peptides were desalted and dried as described above.

### Mass spectrometry analysis

#### LC-MS system for the *S*-palmitoylated peptide mixture

The *S*-palmitoylated peptide solution (20 μg) was dissolved in 80% acetonitrile, 20% DMSO with 0.1% TFA, and 4 pmol was injected per measurement. Peptides were separated on a UHPLC system (Ultimate 3000, Thermo Fisher Scientific) coupled to an Orbitrap Exploris 480 mass spectrometer (Thermo Fisher Scientific) in data-dependent acquisition mode. For C_18_-RP-LC, peptides were loaded onto a Pepmap Neo 100 C_18_ trap column (5 mm × 0.3 mm, 5 μm, Thermo Fisher Scientific) and separated on a homemade analytical column (ReproSil-Pur 120 C_18_-AQ, 50 cm × 75 μm, 2.4 μm, Dr Maisch) at 450 nL min^−1^. For C_4_-RP-LC, peptides were loaded onto a Reprospher 100 C_4_ trap column (5 mm × 0.3 mm, 5 μm, Dr Maisch) and separated on a homemade analytical column (ReproSil Gold 120 C_4_, 50 cm × 75 μm, 3 μm, Dr Maisch) at 450 nL min^−1^. Solvent A is 0.1% formic acid and solvent B is 85% acetonitrile, 15% isopropanol with 0.1% formic acid. Other settings are specified in the results. A 30 min gradient was applied: 9% solvent B for 1 min, 9–25% for 1 min, 25–99% for 12 min, 99% for 5 min, 99–9% for 1 min and 9% for 10 min. MS1 scans were performed at 60 000 resolution between 375 and 2000 *m*/*z* after reaching the normalised AGC target with automatic injection time every second.

#### MS/MS fragmentation of the *S*-palmitoylated peptide mixture

The *S*-palmitoylated peptide solution (20 pmol per measurement) was analysed on a UHPLC system (optimised C_18_-RP-LC, 30 min gradient described above) coupled to an Orbitrap Fusion Lumos Tribrid mass spectrometer (Thermo Fisher Scientific) in data-dependent acquisition mode. Precursor ions of interest were isolated by the quadrupole and fragmented with CID, HCD or ETD (with or without supplemental activation) at a resolution of 30 000. Other settings are specified in the results (if not, then standard settings were applied).

#### LC-MS/MS analysis of other samples

The samples were dissolved in 80% acetonitrile, 20% DMSO with 0.1% TFA. The spiked samples were measured on the optimised C_18_-RP-LC system with the same gradient, solvents and MS method as described above for the LC systems; 14 pmol was injected per measurement (calculated based on the spike). The transfected samples (10% injected) were measured on the optimised C_18_-RP-LC system with the same solvents as described above. The peptides were separated with a 180 min gradient: 9% solvent B for 2 min, 9–80% for 161 min, 80–99% for 2 min, 99% for 5 min and 9% for 10 min. MS1 scans were performed at a resolution of 60 000 between 375 and 2000 *m*/*z* after reaching the normalised AGC target with automatic injection time every second. The top intense precursors were fragmented with a normalised HCD collision energy of 28% and 24 s dynamic exclusion time. HCD fragmentation was performed on precursors at a resolution of 30 000.

#### Database search

Raw data were processed with FragPipe v21.1 with MSFragger v4.0, IonQuant v1.10.12 and Philosopher v5.1.0 using the default settings.^38^ For the synthetic peptides, MS/MS spectra were searched against a protein sequence including the modified peptide target (4 entries) with added decoys and common contaminants. MSFragger search included mass calibration with a precursor and fragment mass tolerance of 20 ppm, peptide length between 5 and 50, and *S*-palmitoylation (238.22968, C) as a variable modification. The activation type filter and fragment ion series were altered based on the fragmentation method used. Validation was performed using Percolator with a false discovery rate for PSMs set to 1%. For the spiked samples, MS/MS spectra were searched against the human UniProt database containing the synthetic peptide sequences in their corresponding protein sequences (20 428 entries, version November 2023, UP000005640) with added decoys and common contaminants. MSFragger search included mass calibration with a precursor and fragment mass tolerance of 20 ppm, peptide length between 5 and 50, a strict trypsin enzymatic search with two missed cleavages and protein N-terminal methionine cleavage. The search also included variable modifications: protein N-terminal acetylation, methionine oxidation, *S*-palmitoylation (238.22968, C) and alkylation with NEM (125.047676, C). For the transfection samples, MS/MS spectra were searched against the human UniProt database containing the modified GNA13 or RhoB sequence (20 419 + 3 entries, version May 2024, UP000005640) with added decoys and common contaminants. The MSFragger search included mass calibration with a precursor and fragment mass tolerance of 20 ppm, peptide length between 7 and 50, a strict trypsin enzymatic search with two missed cleavages and protein N-terminal methionine cleavage. The search also included variable modifications: protein N-terminal acetylation, methionine oxidation, myristoylation (210.19836, C[^), palmitoylation (238.22968, C[^), stearoylation (266.26096, C[^), farnesylation (204.1878, C), geranylgeranylation (272.2504, C) and carbamidomethylation. Validation was performed using PeptideProphet, and ProteinProphet was used for protein inference with the false discovery rate set to 1%.

#### Data processing and visualisation

Peaks of the synthetic *S*-palmitoylated peptides were selected in the raw chromatogram with FreeStyle™ 1.8 SP2 QF1 using mass range. MS/MS spectra were visualised using FragmentLab v2.6.1.32. Ion coverages were calculated with an in-house script developed using Rust and rustyms (https://github.com/snijderlab/rustyms).^22^ LC peak areas were determined with Skyline v24.1.0.199. All figures were graphed with RStudio version 2024.04.2+764 using R v4.4.0 and further edited with Adobe Illustrator 2024.

## Results and discussion

### Separation of long-chain *S*-acylated peptides by reversed-phase liquid chromatography

**Fig. 1:**
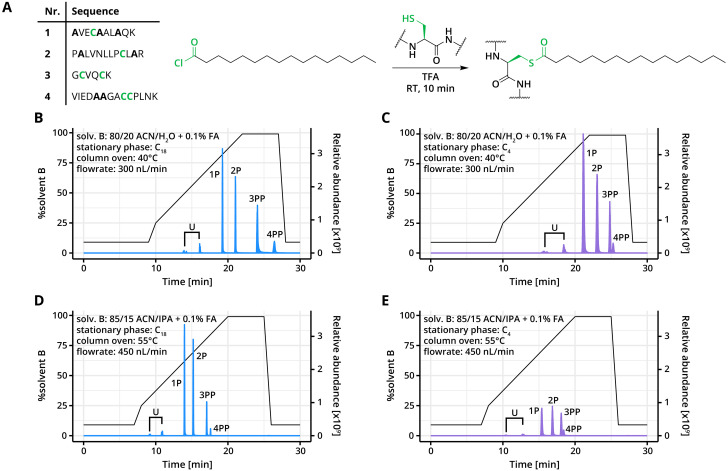
Elution profile of *S*-palmitoylated peptides using different LC strategies. (A) The sequences of the synthetic peptides are shown in the table, with the number representing the sequence. The bolded amino acids represent serine, threonine, or tyrosine residues that were substituted with alanine. Synthetic peptides were reacted with palmitoyl chloride to generate *S*-palmitoylated peptides. (B–E) The *S*-palmitoylated peptide mixture was dissolved in 80% acetonitrile, 20% DMSO with 0.1% TFA and analysed using a (B/D) C_18_ or (C/E) C_4_ trap and column system (2 pmol per injection) with the autosampler temperature set at 21 °C. Measurements were conducted in duplicates. Mobile phase A was 0.1% formic acid. Mobile phase B and additional settings are specified in each panel. The sample contains unmodified peptides 1, 2, and 4 noted together as U; singly *S*-palmitoylated peptides 1P and 2P; and dually *S*-palmitoylated peptides 3PP and 4PP. P stands for one palmitoyl moiety, and PP stands for two palmitoyl moieties attached to the peptide.

Long-chain *S*-acylated peptides are challenging to detect by bottom-up proteomic analysis due to their highly hydrophobic nature and low abundance. When standard LC gradients optimised for unmodified peptides are applied, the long-chain *S*-acylated peptides typically elute at the end of or after the LC gradient, due to their strong affinity for the C_18_ stationary phase. To develop a reversed-phase chromatographic strategy compatible with separating long-chain *S*-acylated peptides, we compared elution profiles on C_4_ or C_18_ stationary phases with various mobile phases. Synthetic tryptic peptides inspired by known *S*-acylated proteins (CDC42, HTT, ZDHHC6 and FYN) were synthesised and used as model peptides (Fig. 1A). These peptides were individually *S*-palmitoylated by reacting with palmitoyl chloride following a previously published protocol.^37^ The were combined to provide a mixture of unmodified, mono- and di-palmitoylated peptides to determine the optimal chromatographic conditions for separating long-chain *S*-acylated peptides.^37^ Of note, to simplify our model peptides, serine, threonine, and tyrosine residues present in the parent peptides of the corresponding proteins were changed to alanine residues to prevent *O*-palmitoylation during *S*-palmitoylation of these peptides.

Initially, the *S*-palmitoylated peptide mixture was analysed on a standard LC-MS system designed for unmodified peptides. This setup employed a C_18_ stationary phase (trap and column) with 0.1% formic acid as mobile phase A and 80% acetonitrile and 0.1% formic acid as mobile phase B. In standard separation strategies, mobile phase B is increased from 10% to 45% to facilitate the separation and elution of unmodified peptides. However, under these conditions, long-chain *S*-acylated peptides remained strongly retained. Extending the gradient to 100% mobile phase B improved the elution of the *S*-palmitoylated peptides (Fig. 1B). Unmodified peptides eluted before 60% B, while mono-palmitoylated peptides 1**P** and 2**P** required 80% B, and di-palmitoylated peptides 3PP and **4PP** only eluted after the gradient ended. This indicates that, even with an extended gradient, effective elution of all the long-chain *S*-acylated peptides was not achievable on this setup.

To enhance the elution of these highly hydrophobic peptides, we evaluated a C_4_ stationary phase with the same mobile phases (Fig. 1C). The use of the C_4_ stationary phase resulted in slightly reduced separation efficiency and produced broader peaks, possibly due to its larger particle size and fewer hydrophobic interactions with the peptides. These two factors affect the mass transfer efficiency, causing higher variability in the elution times. Still, the order of elution and peak intensity remained comparable to the C_18_ stationary phase. Surprisingly, retention times of the peptides on the C_4_ stationary phase were longer than expected for a less hydrophobic phase. This is likely influenced by the particle material, larger packing particles, and lower backpressure, which together resulted in increased diffusion and delayed elution.

As the C_4_ stationary phase did not yield improvements over C_18_, we next modified mobile phase B by including isopropanol to reduce the retention time of long-chain *S*-acylated peptides. We used 15% isopropanol, 85% acetonitrile with 0.1% formic acid as mobile phase B, and additionally raised the column temperature to 55 °C to reduce the viscosity of mobile phase B. These changes significantly reduced retention times for both mono- and di-palmitoylated peptides (Fig. 1D and E) on both C_4_ and C_18_ stationary phases, achieving elution between 60% and 80% B. The C_18_ phase provided sharper peaks and better separation between peptides, which will provide better resolution for complex samples. Notably, unmodified peptides also eluted efficiently within this gradient, confirming that the optimised C_18_-RP-LC system effectively separates a broad range of peptides. Consequently, this optimised C_18_-RP-LC system was selected for subsequent experiments to achieve efficient separation and elution of long-chain *S*-acylated peptides.

### Fragmentation of long-chain *S*-acylated peptides with tandem mass spectrometry

In bottom-up proteomics, tandem mass spectrometry (MS/MS) is a crucial step for identifying modified amino acids within proteins. However, long-chain *S*-acylated peptides, which contain labile thioester bonds, present challenges in PTM identification and localisation following MS/MS fragmentation. To determine the optimal MS/MS fragmentation strategy for long-chain *S*-acylated peptides, we systematically examined the fragmentation behaviour of the synthetic *S*-palmitoylated peptide mixture using collision-induced dissociation (CID), higher-energy collisional dissociation (HCD), and electron transfer dissociation (ETD, with and without supplemental activation) under varying collision energies and ETD reaction times. These analyses were performed using the optimised C_18_-RP-LC strategy coupled to an Orbitrap Fusion Lumos Tribrid mass spectrometer.

**Fig. 2:**
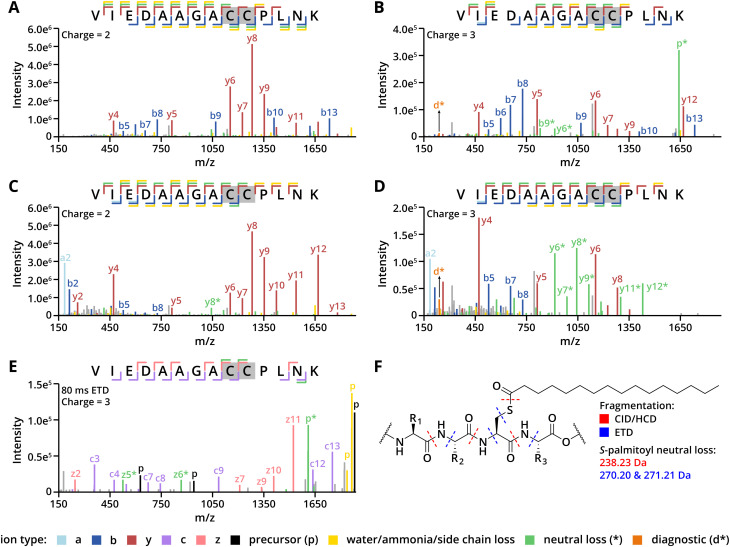
(A, B) MS/MS spectra of peptide 4PP fragmented with CID at collision energy 35%. (C/D) MS/MS spectra of peptide 4PP fragmented with HCD at 28% collision energy. (E) MS/MS spectra of peptide 4PP fragmented with ETD at a reaction time of 80 ms. (F) MS/MS fragmentation pattern of long-chain *S*-acylated peptides.

CID fragmentation of the four *S*-palmitoylated peptides yielded MS/MS spectra dominated by sequence-informative b- and y-ions with high abundance across all collision energies, resulting in high sequence coverage for all peptides (Fig. 2A, B, S2 and S3). For peptides 1P and 4PP, in charge state 2+, we observed low-intensity y-ions with a palmitoyl loss (238.23 Da, C_16_H_30_O); hereafter, ions with neutral losses are indicated with an asterisk [*] (Fig. 2A, F and S2A). These y*-ions were lower in intensity than the b- and y-ions without this neutral loss. For triply charged peptides 1P and 4PP, the precursor ion with palmitoyl loss, hereafter p*-ion, was detected at higher intensities than the b- and y-ions (Fig. 2B and S2B). The sequence coverage of peptide 4PP at charge state 3+ was also lower (Fig. 2B). Additionally, for triply charged peptides, a diagnostic ion specific to the palmitoyl group (239.24 Da, d*-ion) was present (Fig. 2B and S2B). For the doubly charged shorter peptide 3PP, both y*- and p*-ions were prominent in the spectra, with the p*-ion dominating (Fig. S2E). In contrast, peptide 2P did not show any y*- or p*-ions in the CID spectra, suggesting a greater stability of the thioester bond within this sequence (Fig. S2F). Consequently, for doubly charged peptides, CID spectra primarily feature sequence-informative ions, while for higher charge states and shorter peptides, palmitoyl loss dominates, potentially affecting sequence coverage.

HCD fragmentation, in contrast, produced spectra with highly abundant y-ions for both doubly and triply charged states, while b-ions were predominantly present in the low-mass region (Fig. 2C, D and S4). Nonetheless, high sequence coverage was maintained for each peptide, which only decreased at collision energies above 30% (Fig. S5). For peptides **2P** and **3PP**, the fragmentation behaviour with HCD is similar to that observed with CID (Fig. S4E and S4F). Interestingly, for peptides 1P and 4PP, HCD generated y*-ions instead of p*-ions in both charge states, with y*-ions for the triply charged peptides reaching intensities comparable to the sequence-informative y-ions (Fig. 2C, D, S4A and S4B). This shift to y*-ions over p*-ions in HCD compared to CID spectra is advantageous as y*-ions retain sequence information, unlike the predominant p*-ions in CID spectra.

Next, ETD fragmentation was explored for its compatibility with long-chain *S*-acylation detection, as it has shown promise in the analysis of other PTMs like phosphorylation and glycosylation.^39,40^ ETD preferentially cleaves N–Cα bonds along the peptide backbone, which theoretically preserves the thioester bond in long-chain *S*-acylated peptides. We fragmented the *S*-palmitoylated peptide mixture using ETD with varied reaction times (Fig. 2E, S6 and S7). For peptides 1P, 2P, and 4PP, ETD produced c- and z˙-ions, resulting in high sequence coverage (Fig. 2E and S6). However, while the thioester bond remained intact, fragmentation of the cysteine side chain produced 270.20 Da (C_16_H_30_OS˙) and 271.21 Da (C_16_H_31_OS˙) losses, though the d*-ion was absent (Fig. 2F). The precursor ions with ±H_2_O or palmitoylation loss were prevalent, as also seen with CID (Fig. 2E and S6). Especially with peptide 3**PP**, only precursor ions with neutral losses were generated. For this reason, peptide 3**PP** was not identified in database searches with Fragpipe under any ETD conditions, indicating that shorter *S*-acylated peptides might be challenging to sequence with ETD. Reducing p*-ion intensity was possible by extending the ETD reaction time, although this did not consistently improve the c- and z˙-ion coverage (Fig. S7A–C). Additionally, testing ETD with supplemental activation showed no significant improvement for peptides 1P, 2P, and 4PP (Fig. S8A–D, S8G, S8H and S9). However, it enhanced fragmentation of peptide 3PP, yielding a spectrum suitable for sequence determination and showed that ultimately CID and HCD are better suited for shorter long-chain *S*-acylated peptides (Fig. S8E and S8F).

While the efficiency of fragmentation and fragmentation patterns are influenced by peptide length, charge, sequence, and the selected fragmentation method, all tested methods successfully detect long-chain *S*-acylated peptides. It is important to note that most spectra were dominated by p*-, y*- and z*-ions, demonstrating that across these fragmentation techniques, long-chain *S*-acylation consistently cleaves during fragmentation, which is in line with previous studies.^31,36^ Thioester bond fragmentation is induced with CID and HCD, while ETD cleaves the S–C bond within the cysteine side chain (Fig. 2F). With CID and ETD, the modification is mostly cleaved from the precursor ion (= p*-ion), which depending on the peptide may affect its sequence coverage. In contrast, neutral loss from y-ions is predominant in HCD. As these secondary fragment ions also provide sequence information, HCD revealed to be the most effective at sequencing the four peptides.

### Stability of long-chain *S*-acylated peptides during bottom-up proteomics sample preparation

Thioesters are known for their chemical instability, which makes them highly reactive functional groups. This inherent instability of thioester bonds poses a significant challenge to the detection of long-chain *S*-acylated peptides.^31,41^ To address this issue, we investigated the stability of synthetic *S*-palmitoylated peptides by monitoring changes in their MS intensity, which could potentially be affected by hydrolysis during bottom-up proteomics sample preparation. Synthetic *S*-palmitoylated peptides were spiked into a protein digest either before or after exposure to the test conditions, and their relative abundances were quantified using MS1 peak area integration.

**Fig. 3:**
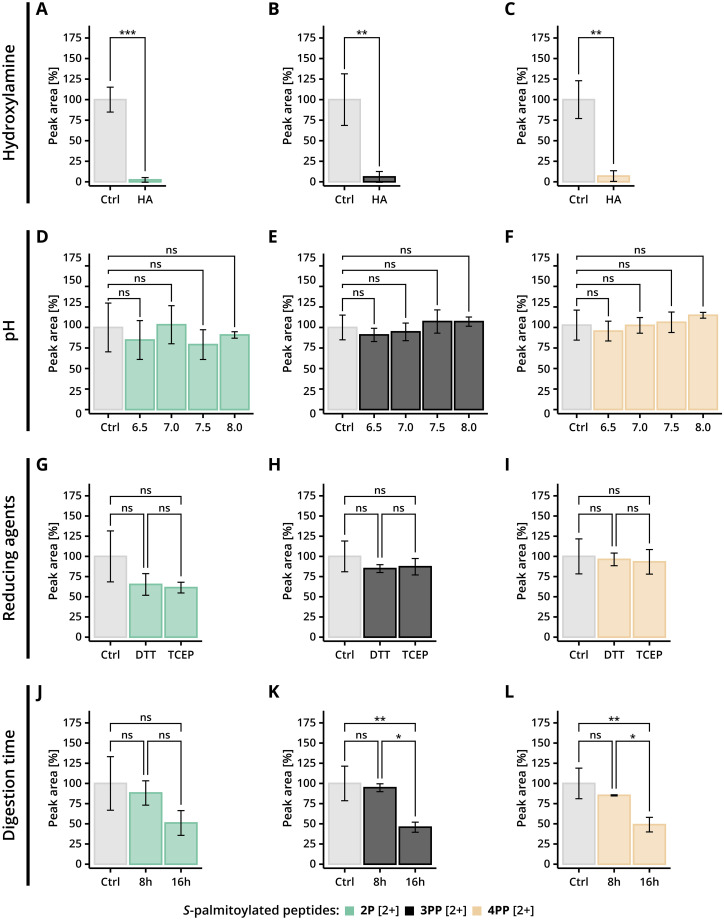
Stability of *S*-palmitoylated peptides following various treatments. (A–C) *S*-Palmitoylated peptides 2–4 were incubated with hydroxylamine at pH 7.5 for 1 h at RT (*n* = 3). (D–F) *S*-Palmitoylated peptides 2–4 were incubated at different pH for 2 h at RT. Control samples for individual conditions were combined as the spike is the same for each experimental condition (*n*_cntrl_ = 12, *n*_exp_ = 3). (G–I) *S*-Palmitoylated peptides 2–4 were incubated with 10 mM reducing agents at pH 7.5 for 1 h at RT. Control samples for individual conditions were combined as the spike is the same for each experimental condition (*n*_cntrl_ = 6, *n*_exp_ = 3). (J–L) *S*-Palmitoylated peptides 2–4 were incubated with trypsin in 50 mM HEPES pH 7.5 for 8 h or 16 h at 37 °C. Control samples for individual conditions were combined as the spike is the same for each experimental condition (*n*_cntrl_ = 6, *n*_exp_ = 3). Peak areas from the chromatogram were normalised to control values, and adjusted *p*-values were calculated using either (A–C) Student's unpaired *t*-test or (D–L) one-way ANOVA test with Tukey's multiple comparisons test. Significance levels are indicated as follows: ns = not significant, *p ≤ 0.05, **p ≤ 0.01, ***p ≤ 0.001. Data represent mean ± SD.

To validate our stability assay, we employed hydroxylamine (HA) as a positive control. HA is widely used in long-chain *S*-acylation studies because of its proven ability to selectively cleave thioester bonds, generating free thiols and fatty acid hydroxamates.^28,42,43^*S*-Palmitoylated peptides were incubated with 1 M HA at RT for 1 hour. As expected, *S*-palmitoylated peptides 2–4 exhibited significant palmitic acid loss after incubation (Fig. 3A–C). Surprisingly, peptide 1P displayed resistance to HA cleavage (Fig. S10A). While HA treatment is generally efficient at cleaving thioester bonds, some peptides may exhibit resistance due to their primary sequence and/or conformational properties. Nonetheless, *S*-palmitoylated peptides 2–4 showed a clear and expected response to HA treatment, validating its use in assays designed to study the hydrolysis of long-chain *S*-acylated peptides.

This assay was first employed to assess the stability of *S*-palmitoylated peptides across various pH levels, aiming to determine buffer conditions compatible with proteomics workflows. *S*-Palmitoylated peptides were incubated in HEPES-buffered solutions at different pH values for 1 hour. Notably, no significant loss of palmitic acid was observed at any tested pH (Fig. 3D–F and S10B).

Next, we examined the effect of different reducing agents on thioester stability. Dithiothreitol (DTT) is a commonly used reducing agent with two thiol groups and can act as a nucleophile toward thioesters. In contrast to DTT, tris(2-carboxyethyl)phosphine (TCEP) is generally considered less reactive toward thioesters; however, a potential mechanism targeting acylated cysteines has recently been reported.^44^ Therefore, selecting an appropriate reducing agent is critical when studying long-chain *S*-acylation. We incubated the *S*-palmitoylated peptides with DTT or TCEP at RT for 1 hour. None of the four *S*-palmitoylated peptides demonstrated significant *S*-palmitoyl loss following treatment with either reducing agent (Fig. 3G–I and S10C). The observed thioester stability with TCEP aligns with existing literature.^31^ However, previous studies have reported significant palmitic acid loss during DTT treatment at 37 °C.^31^ The discrepancy between these results may be attributed to the higher incubation temperature in earlier studies compared to our incubation with DTT at RT, suggesting that temperature sensitivity plays a role in the variability observed across experiments.^31^

Lastly, we assessed thioester stability during trypsin digestion, a critical step in bottom-up proteomics workflows. *S*-Palmitoylated peptides were incubated at 37 °C with trypsin for 8 and 16 hours (Fig. 3J–L and S10D). Our findings indicate that all peptides remained stable during 8 hours of digestion. However, after 16 hours of prolonged digestion, a loss of signal was observed for all *S*-palmitoylated peptides. In particular, the di-palmitoylated peptides 3**PP** and 4**PP** displayed a significant loss of the *S*-palmitoylated peptide signal. This loss of signal may be caused by thioester hydrolysis, the precipitation of *S*-palmitoylated peptides out of solution, or a combination thereof. In conclusion, our data demonstrate that *S*-palmitoylated peptides are stable in HEPES buffer at pH levels up to 8.0 and under reducing conditions, but prolonged tryptic digestion can lead to substantial loss of *S*-palmitoylation.

### Direct detection of long-chain *S*-acylated peptides in a complex proteome

After optimising parameters for the direct detection of long-chain *S*-acylated peptides, we applied our bottom-up proteomics workflow to analyse endogenous long-chain *S*-acylation in proteins. To this end, we transfected HEK293T cells with GNA13, a known long-chain *S*-acylated protein that encodes one of the alpha subunits (GNA13/Gα13) of heterotrimeric G proteins, which mediate signal transduction from G protein-coupled receptors (GPCRs).^45,46^ Long-chain *S*-acylation is essential for the association of GNA13 with the plasma membrane and the subsequent activation of Rho-dependent signalling pathways. Previous studies employing indirect techniques, such as acyl-biotin exchange and [^3^H]-palmitic acid labelling, have demonstrated *S*-palmitoylation at cysteine residues 14 and 18 of GNA13.^45,46^

**Fig. 4:**
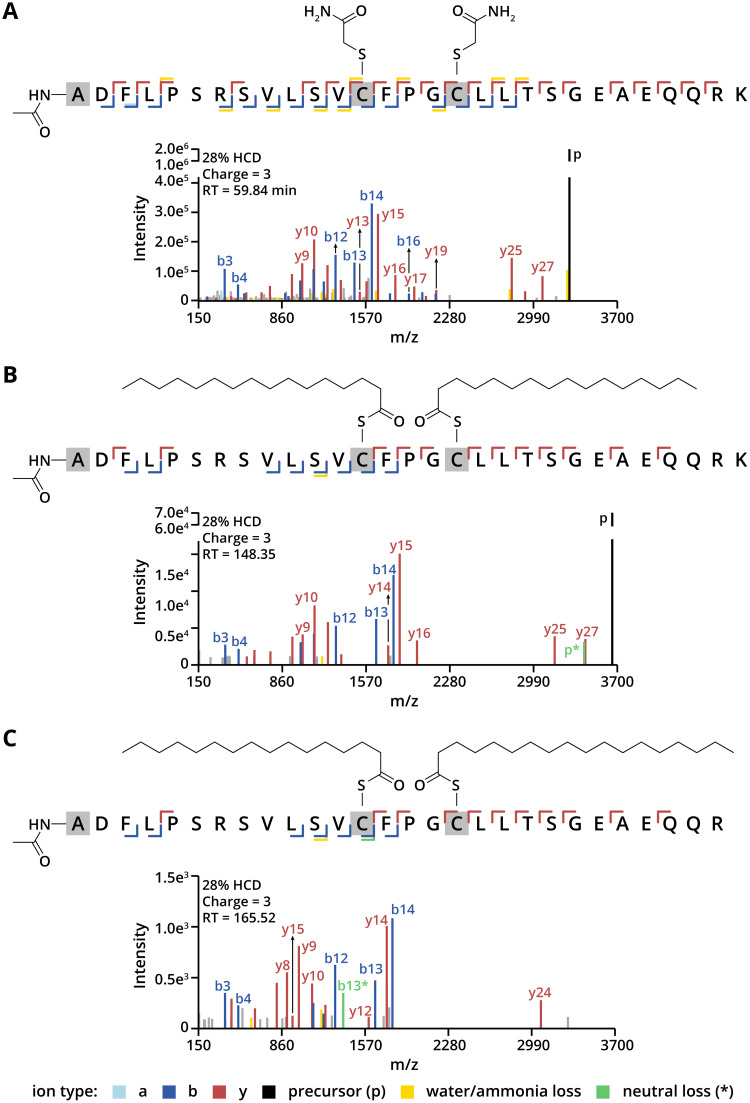
(A) MS/MS spectra of unmodified GNA13 peptide fragmented with HCD. (B) MS/MS spectra of dually *S*-palmitoylated GNA13 peptide fragmented with HCD. (C) MS/MS spectra of cys14-palmitoylated/cys18-stearoylated GNA13 peptide fragmented with HCD.

In our analysis, we detected two long-chain *S*-acylation sites within the same tryptic peptide and identified two distinct long-chain *S*-acylated peptidoforms. In one form, both Cys14 and Cys18 were palmitoylated (Fig. 4B), while in the other form, Cys14 carried a palmitoyl group, and Cys18 was stearoylated (Fig. 4C). These two long-chain *S*-acylated peptidoforms of GNA13 may serve distinct functional roles and require further investigation. Interestingly, HCD fragmentation of these di-acylated GNA13 peptides did not result in neutral losses or diagnostic ions, as observed for the synthetic *S*-palmitoylated peptides. This difference may originate from peptide-specific properties. To optimise fragmentation of longer long-chain *S*-acylated peptides alongside shorter ones, a stepped HCD method may be beneficial. Our direct analysis also detected unmodified GNA13 with both Cys14 and Cys18 identified as carbamidomethylated (Fig. 4A). The difference in abundance between unmodified and *S*-acylated GNA13 peptides may result from the sub-stoichiometric levels of *S*-acylation in transiently expressed GNA13. Alternatively, it could be due to the loss of both *S*-acyl groups during digestion or could be an artifact of GNA13 overexpression. Notably, we did not detect a mono-acylated form, suggesting that the second acylation occurs rapidly following the initial modification.

**Fig. 5:**
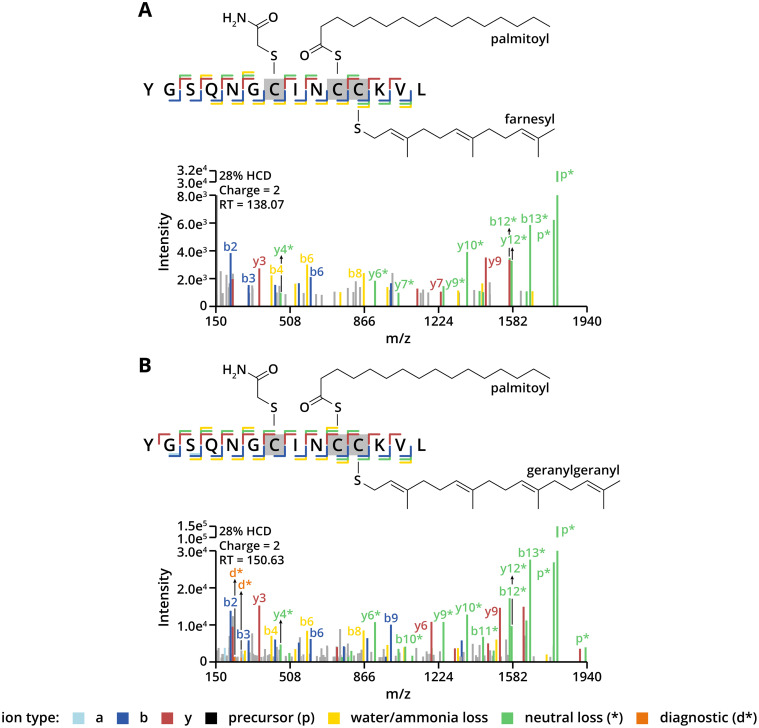
(A) MS/MS spectra of *S*-palmitoylated/farnesylated RhoB peptide fragmented with HCD. (B) MS/MS spectra of *S*-palmitoylated/geranylgeranylated RhoB peptide fragmented with HCD.

Additionally, we investigated the long-chain *S*-acylation of RhoB, a Rho family GTPase involved in regulating the actin cytoskeleton, cell survival, and gene expression.^47^ Long-chain *S*-acylation of RhoB at Cys192 is critical for its tumour suppressor function.^47^ Besides long-chain *S*-acylation, RhoB is known to be prenylated at Cys193; however, these lipid modifications have only been indirectly characterised in previous studies, with their identification largely relying on indirect biochemical techniques. In our study, direct detection of the C-terminal peptide from RhoB confirmed that Cys192 is exclusively long-chain *S*-acylated by palmitic acid (Fig. 5). Cys189 could theoretically serve as a long-chain *S*-acylation site, but we found no evidence for its modification, aligning with reports that this residue is not crucial for RhoB's function or localisation.^47^

The prenylation status of RhoB is associated with its subcellular localisation: geranylgeranylated RhoB is typically found in late endosomes, whereas farnesylated RhoB localises to the plasma membrane.^47,48^ Our analytical strategy not only allowed analysis of long-chain *S*-acylation but also enabled the assessment of RhoB's prenylation status. Specifically, we observed that Cys193 is modified with either a farnesyl or geranylgeranyl group and detected them at significantly different retention times (Fig. 5). Interestingly, neutral losses derived from y-, b- and p-ions were highly abundant in the spectra, which were only derived from farnesylated (204.19 Da, C_15_H_24_) and geranylgeranylated cysteines (272.25 Da, C_20_H_32_). This suggests that, during fragmentation, the thioether bond is less stable than the thioester bond but also promotes p*-ion formation, which was more common in ETD and CID, as shown for the synthetic *S*-palmitoylated peptides. Additionally, a 136.14 Da neutral loss (C_10_H_16_) from the precursor ion was found, which indicates fragmentation of the geranylgeranyl moiety. Furthermore, all detected C-terminal peptides had the carboxyl-terminal sequence KVL, suggesting that RhoB had not undergone post-prenylation processing (specifically, the endoproteolytic removal of the C-terminal AAX motif and carboxymethylation). However, this does not eliminate the possibility of processed and carboxymethylated RhoB, as such a processed RhoB peptide (YGSQNGCINCC) may be challenging to detect post-trypsin digestion due to reduced charge.

In addition to the lipidated peptides from the overexpressed proteins GNA13 and RhoB in HEK293T digests, we detected *N*-myristoylated peptides derived from proteins ADP-ribosylation factor 4 (ARF4), NADH-cytochrome b5 reductase 3 (CYB5R3) and holocytochrome c-type synthase (HCCS), and prenylated peptides from Ras-related proteins RAB11A, RAB11B, RAB1A, RAB1B, RAB2A, RAB5A and RAB5C (SI Table 1 and Fig. S11). Long-chain *S*-acylated peptides from proteins other than the overexpressed RhoB and GNA13 were not detected. This may originate from a lower abundance of *S*-acylated peptides compared to the irreversibly modified *N*-myristoylated and prenylated peptides.

## Conclusion

Long-chain *S*-acylation has been described as a labile, hydrophobic modification, which is difficult to directly detect with a standard bottom-up proteomics workflow. Our optimised bottom-up proteomics workflow successfully addresses several of the challenges associated with the direct detection of long-chain *S*-acylated peptides. Through systematic LC optimisation, we achieved enhanced separation and elution of the hydrophobic long-chain *S*-acylated peptides on a C_18_ stationary phase. Long-chain *S*-acylated peptides were also shown to be stable at various pH levels and reducing conditions, although extended trypsin digestion posed limitations, especially for di-acylated peptides. We demonstrated that the fragmentation methods CID, HCD and ETD can be applied to sequence long-chain *S*-acylated peptides. Across all these fragmentation methods, neutral losses of the modification occurred from the sequence-informative fragment ions (b-, y- and z˙-ions) and/or precursor ions. However, HCD fragmentation was the most effective approach for preserving the sequence information of long-chain *S*-acylated peptides. Applying this approach to transiently overexpressed GNA13 and RhoB, we differentiated lipid-specific dual lipidation states on GNA13 and directly characterised the long-chain *S*-acylation and prenylation of RhoB.

Additionally, we detected multiple endogenous *N*-myristoylated and prenylated peptides from complex HEK293T digests. While our mass spectrometry-based proteomics workflow provides an approach for characterising long-chain *S*-acylation, our direct-detection workflow falls short of characterising endogenous *S*-acylated sites. However, our optimised methodology shows promise when the protein of interest is over-expressed. As long-chain *S*-acylated proteins represent only a small fraction of the proteome, enrichment strategies for lipidated peptides will be required to allow for proteome-scale analysis of long-chain *S*-acylation. The future integration of an enrichment strategy with our proteomics workflow presents a promising path forward towards proteome-scale analysis of long-chain *S*-acylation with lipid-specific resolution. This combined approach holds significant potential for advancing our understanding of how these hydrophobic modifications regulate protein function and cellular signalling.

## Abbreviations

ABE: Acyl-biotin exchange
ABHD17: α/β hydrolase domain-containing protein 17
ACN: Acetonitrile
AGC: Automatic gain control
APT1/2: Acyl-protein thioesterase 1 and 2
BCA: Bicinchoninic acid
CID: Collision-induced dissociation
CDC42: Cell division control protein 42 homolog
DDM: *n*-Dodecyl-β-d-maltopyranoside
DMSO: Dimethyl sulfoxide
DTT: Dithiothreitol
EDTA: Ethylenediaminetetraacetic acid
ETD: Electron transfer dissociation
FYN: Tyrosine-protein kinase Fyn
GNA13: Guanine nucleotide-binding protein subunit alpha-13
GPCRs: G protein-coupled receptors
HA: Hydroxylamine
HCD: High-energy collisional dissociation
HTT: Huntingtin
IPA: Isopropanol
LC-MS/MS: Liquid chromatography tandem mass spectrometry
LML: Lipid metabolic labelling
MQ: Milli-Q
MS/MS: Tandem mass spectrometry
NEM: *N*-Ethyl maleimide
o/n: Overnight
PATs: Protein acyltransferases
PMSF: Phenylmethylsulphonyl fluoride
PPT1/2: Palmitoyl protein thioesterase 1
PSMs: Peptide-spectrum matches
PTM: Post-translational modification
RhoB: Rho-related GTP-binding protein
RP-LC: Reversed-phase liquid chromatography
RT: Room temperature
SDS: Sodium dodecyl sulphate
TCEP: Tris(2-carboxyethyl)phosphine hydrochloride
TFA: Trifluoroacetic acid
ZDHHC6: Zinc finger DHHC domain-containing protein 6

## Conflicts of interest

There are no conflicts to declare.

## Supplementary Material

AN-150-D5AN00557D-s001

AN-150-D5AN00557D-s002

## Data Availability

Raw data and search results pertaining to the project have been deposited in the ProteomeXchange Consortium *via* the PRIDE partner repository (PXD057394). The data supporting this article and supplementary figures have been included as part of the SI. Fig. S1 Sequences of synthetic *S*-palmitoylated peptides. Fig. S2 MS/MS spectra of *S*-palmitoylated peptides fragmented with CID. Fig. S3 Ion coverages of each *S*-palmitoylated peptide fragmented with CID. Fig. S4 MS/MS spectra of *S*-palmitoylated peptides fragmented with HCD. Fig. S5 Ion coverages of *S*-palmitoylated peptides fragmented with HCD. Fig. S6 MS/MS spectra of *S*-palmitoylated peptides fragmented with ETD. Fig. S7 Ion coverages of *S*-palmitoylated peptides fragmented with ETD. Fig. S8 MS/MS spectra of *S*-palmitoylated peptides fragmented with ETciD and EThcD. Fig. S9 Ion coverages of *S*-palmitoylated peptides fragmented with ETciD and EThcD. Fig. S10 Bar chart of peptide 1 treated at different conditions. Fig. S11 MS/MS spectra of *N*-myristoylated ARF4 and geranylgeranylated RAB5C peptides fragmented with HCD. Supplementary information is available. See DOI: https://doi.org/10.1039/d5an00557d.
