# Supplementary material for: A mass spectrometry-based proteomics strategy to detect long-chain S-acylated peptides
Source: Analyst. 2025 Aug 28;150(19):4367–80. doi: 10.1039/d5an00557d (PMC12394793; doi:10.1039/d5an00557d)
Supplement: AN-150-D5AN00557D-s002 [file AN-150-D5AN00557D-s002.pdf]

Supplementary Information (SI) for Analyst.
This journal is © The Royal Society of Chemistry 2025

Supporting information

A mass spectrometry-based proteomics strategy to detect long-chain *S*-acylated peptides

Samiksha Sardana[1,2], Andrea Trezza[1,2], Francine Rodrigues Ianiski[1,2], Anneroos E. Nederstigt[1,2], Marc P. Baggelaar[1,2,]*

1. Biomolecular Mass Spectrometry and Proteomics, Bijvoet Center for Biomolecular Research and Utrecht Institute for Pharmaceutical Sciences, University of Utrecht, Padualaan 8, Utrecht 3584 CH, The Netherlands

2. Netherlands Proteomics Center, Padualaan 8, Utrecht 3584 CH, The Netherlands

[*] Contact details for correspondence: m.p.baggelaar@uu.nl

Supporting figures

Figure S1. Sequences of synthetic *S*-palmitoylated peptides.

Figure S2. MS/MS spectra of *S*-palmitoylated peptides fragmented with CID.

Figure S3. Ion coverages of each *S*-palmitoylated peptide fragmented with CID.

Figure S4. MS/MS spectra of *S*-palmitoylated peptides fragmented with HCD.

Figure S5. Ion coverages of *S*-palmitoylated peptides fragmented with HCD.

Figure S6. MS/MS spectra of *S*-palmitoylated peptides fragmented with ETD.

Figure S7. Ion coverages of *S*-palmitoylated peptides fragmented with ETD.

Figure S8. MS/MS spectra of *S*-palmitoylated peptides fragmented with ETciD and EThcD.

Figure S9. Ion coverages of *S*-palmitoylated peptides fragmented with ETciD and EThcD.

Figure S10. Bar chart of peptide **1** treated at different conditions.

Figure S11. MS/MS spectra of *N*-myristoylated ARF4 and geranylgeranylated RAB5C peptides fragmented with HCD.

Nr.	Sequence
1	**A**VE**CA**AL**A**QK
2	P**A**LVNLLP**C**L**A**R
3	G**C**VQ**C**K
4	VIED**AA**GA**CC**PLNK

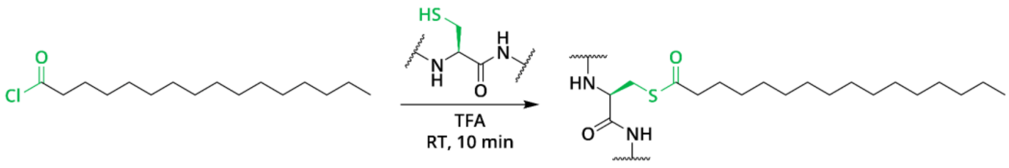

Supporting Figure 1. The sequences of the synthetic peptides are in the table with the number representing the sequence. The bolded amino acids represent serine, threonine, or tyrosine residues that were substituted with alanine. Synthetic peptides were reacted with palmitoyl chloride to generate *S*-palmitoylated peptides.

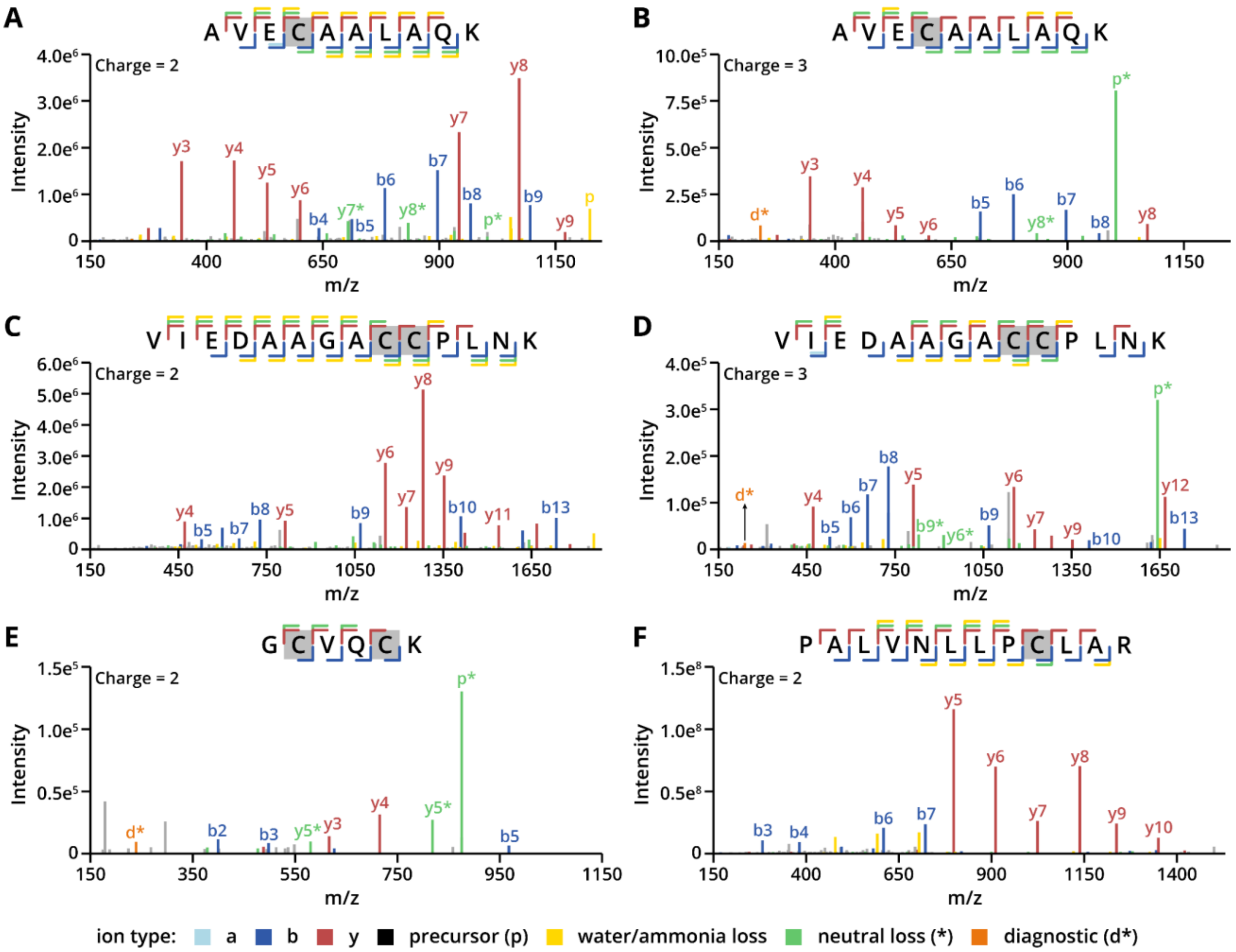

Supporting Figure 2. MS/MS spectra of (A/B) peptide **1P**, (C/D) peptide **4PP**, (E) peptide **3PP** and (F) peptide **2P** obtained using CID fragmentation (collision energy: 35%).

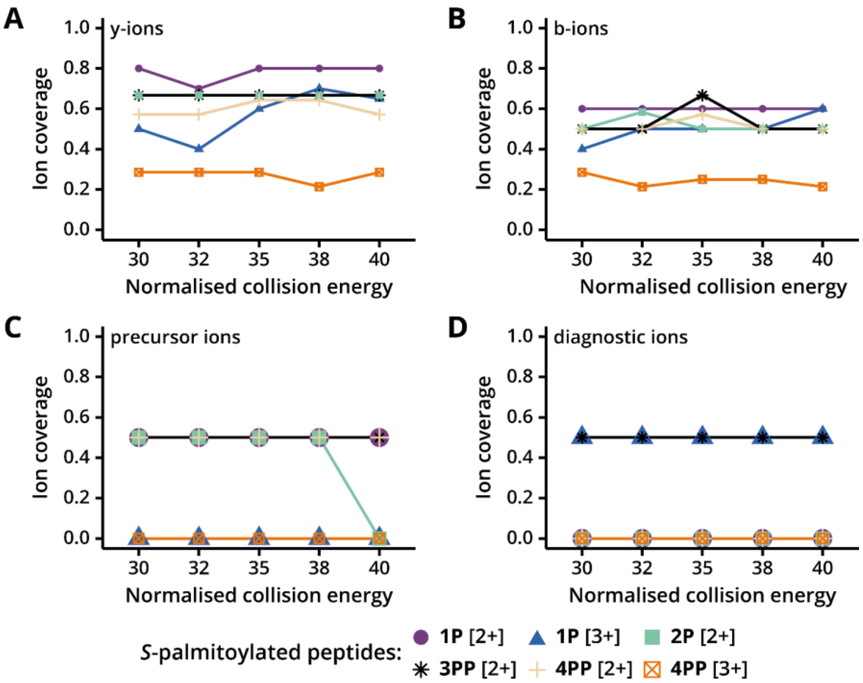

Supporting Figure 3. Ion coverages of (A) y-ions, (B) b-ions, (C) precursor ions, and (D) diagnostic ions of each *S*-palmitoylated peptide fragmented with CID. The y-axis represents the median ion coverage, which is calculated as the total number of experimental ions present in an MS/MS spectrum divided by the total number of theoretical ions.

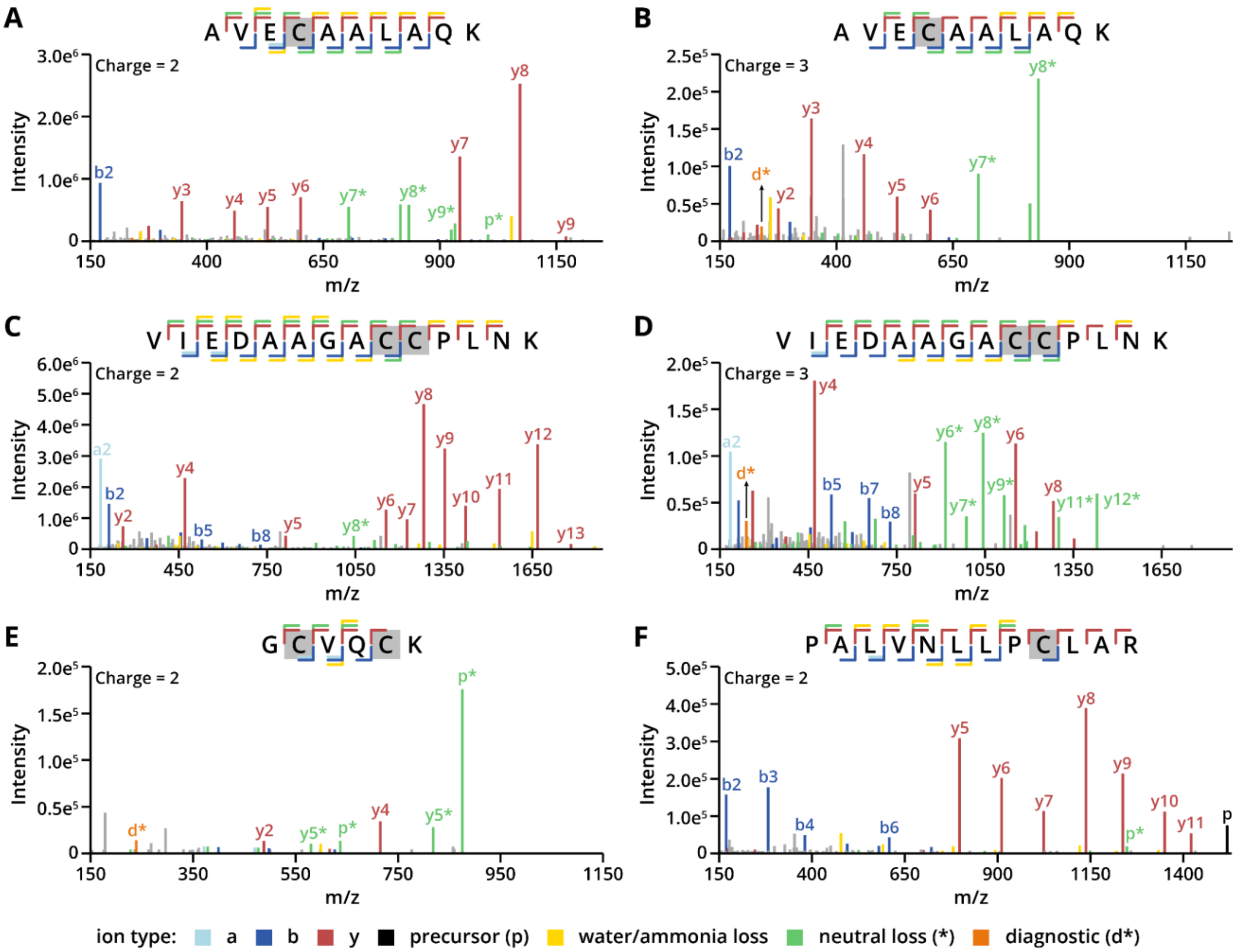

Supporting Figure 4. MS/MS spectra of (A/B) peptide **1P**, (C/D) peptide **4PP**, (E) peptide **3PP** and (F) peptide **2P** obtained using HCD fragmentation (collision energy: 28%).

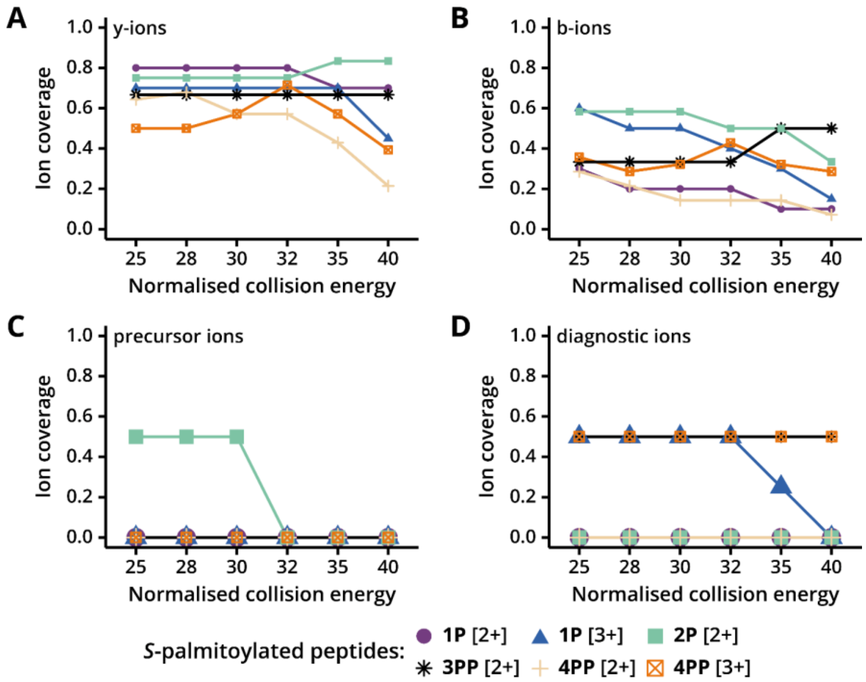

Supporting Figure 5. Ion coverages of (A) y-ions, (B) b-ions, (C) precursor ions, and (D) diagnostic ions of each *S*-palmitoylated peptide fragmented with HCD. The y-axis represents the median ion coverage, which is calculated as the total number of experimental ions present in an MS/MS spectrum divided by the total number of theoretical ions.

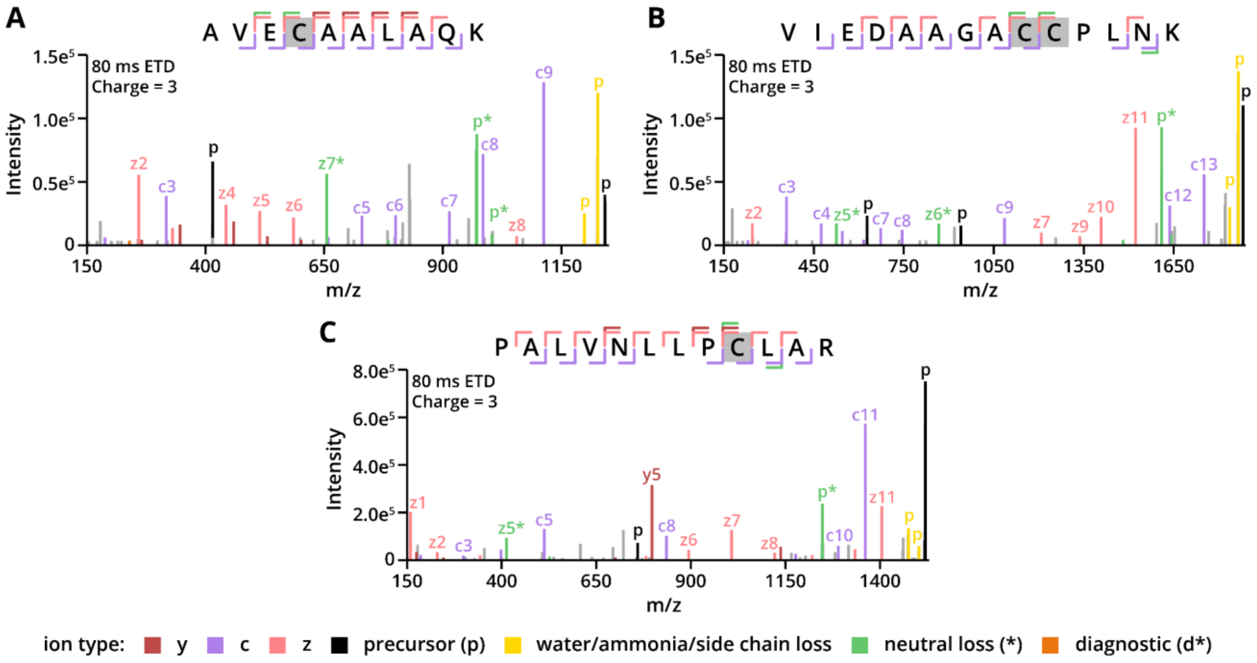

Supporting Figure 6. MS/MS spectra of (A) peptide **1P**, (B) peptide **4PP** and (C) peptide **2P** obtained using ETD fragmentation (reaction time: 80 ms).

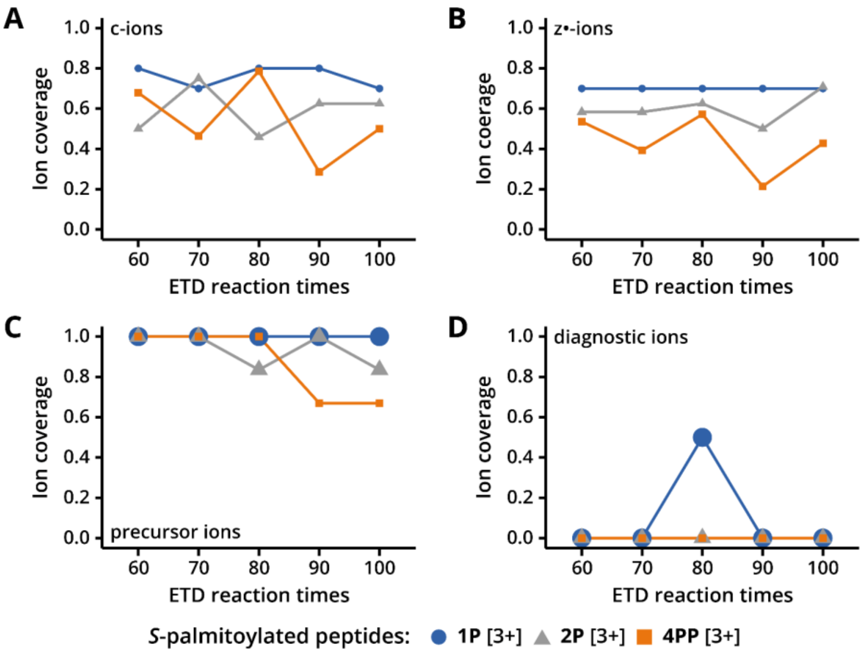

Supporting Figure 7. Ion coverages of (A) y-ions, (B) b-ions, (C) precursor ions, and (D) diagnostic ions of each *S*-palmitoylated peptide fragmented with ETD. The y-axis represents the median ion coverage, which is calculated as the total number of experimental ions present in an MS/MS spectrum divided by the total number of theoretical ions.

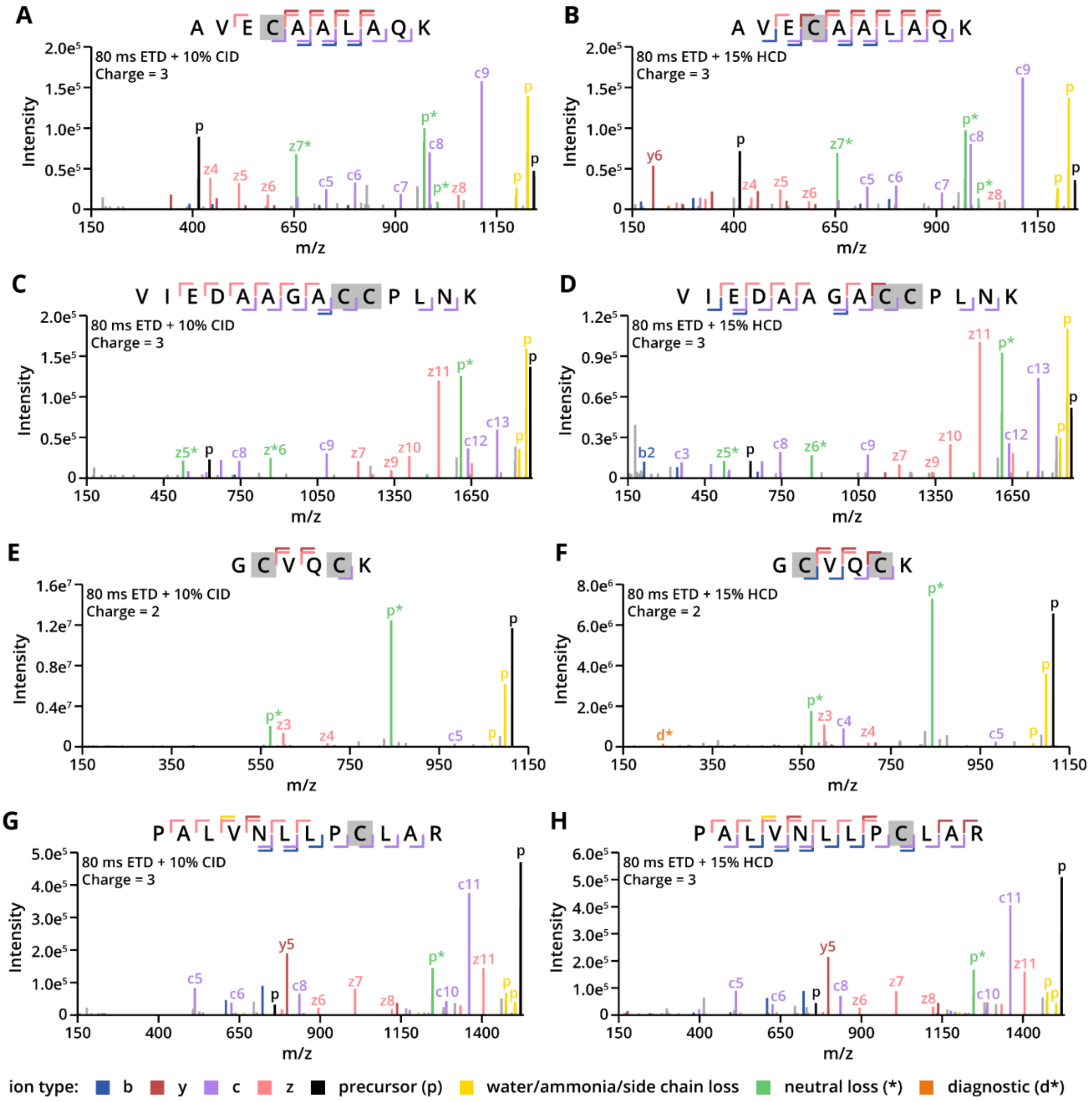

Supporting Figure 8. MS/MS spectra of (A/B) peptide **1P**, (C/D) peptide **4PP**, (E/F) peptide **3PP** and (G/H) peptide **2P** obtained using ETciD/EThcD fragmentation (indicated in the panel).

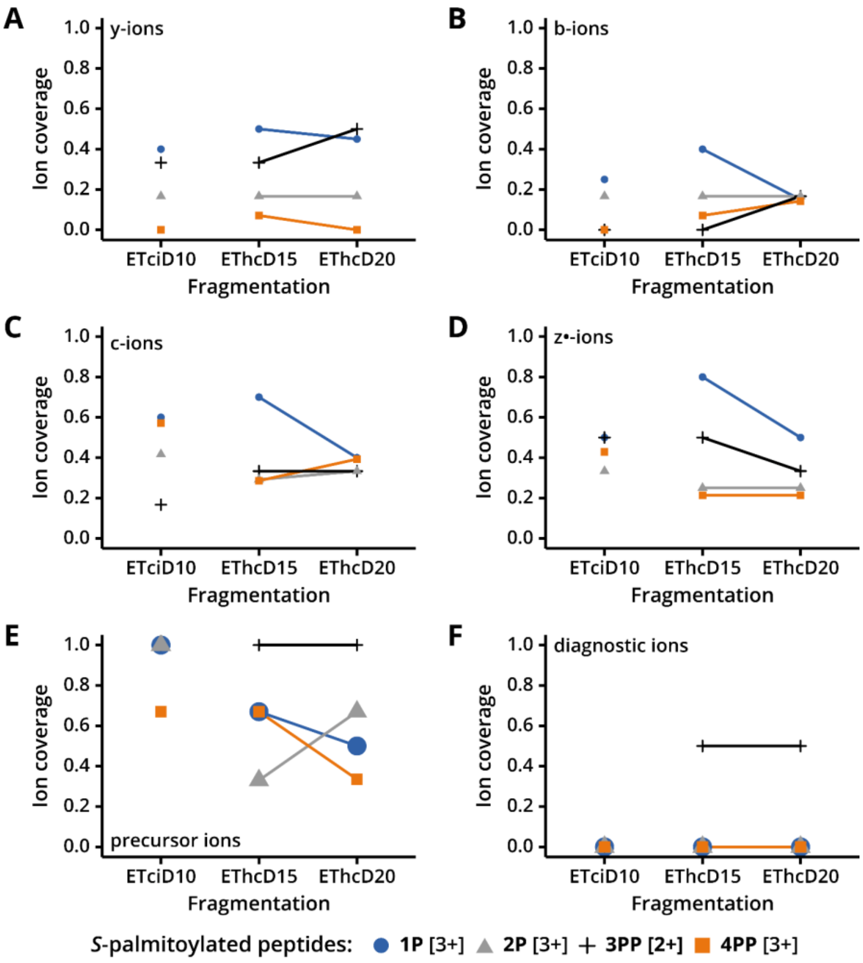

Supporting Figure 9. Ion coverages of (A) y-ions, (B) b-ions, (C) c-ions, (D) z•-ions, (E) precursor ions, and (F) diagnostic ions of each *S*-palmitoylated peptide fragmented with ETciD or EThcD (with the collision energy indicated). The y-axis represents the median ion coverage, which is calculated as the total number of experimental ions present in an MS/MS spectrum divided by the total number of theoretical ions.

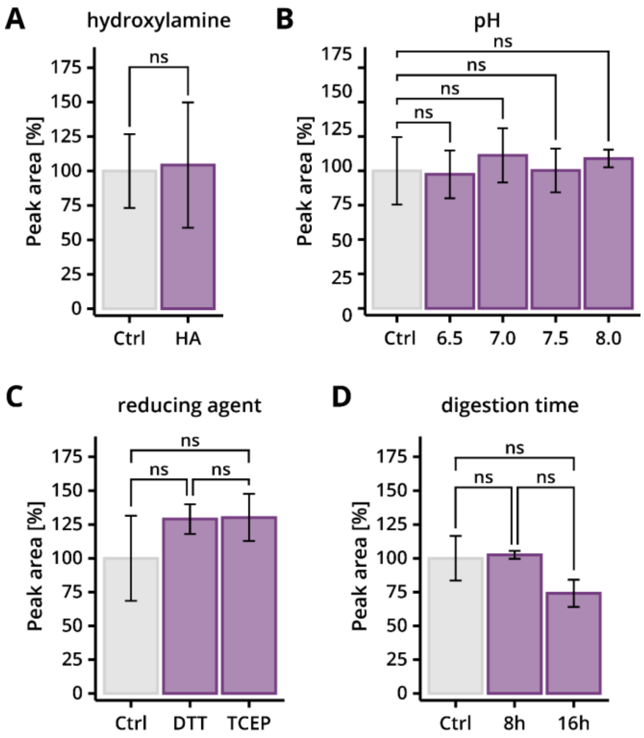

Supporting Figure 10. Stability of *S*-palmitoylated peptides following various treatments. (A) Peptide **1P** was incubated with 1 M hydroxylamine at pH 7.5 for 1h at RT (*n*=3). (B) Peptide **1P** was incubated at different pH for 2h at RT. The control samples for each condition showed similar results, so they were combined (n_{cntrl}=12, n_{exp}=3). (C) Peptide **1P** was incubated with 10 mM reducing agents at pH 7.5 for 1h at RT. The control samples for each condition showed similar results, so they were combined (n_{cntrl}=6, n_{exp}=3). (D) Peptide **1P** was incubated with trypsin in 50 mM HEPES pH 7.5 for 8h or 16h at 37°C. The control samples for each condition showed similar results, so they were combined (n_{cntrl}=6, n_{exp}=3). Peak areas from the chromatogram were normalised to control values, and adjusted *p*-values were calculated using either (A) Student's unpaired *t*-test or (B-D) one-way ANOVA test with Tukey's multiple comparisons test. Significance levels are indicated as follows: ns = not significant, *$p\leq0.05$, **$p\leq0.01$, ***$p\leq0.001$. Data represent mean ± SD.

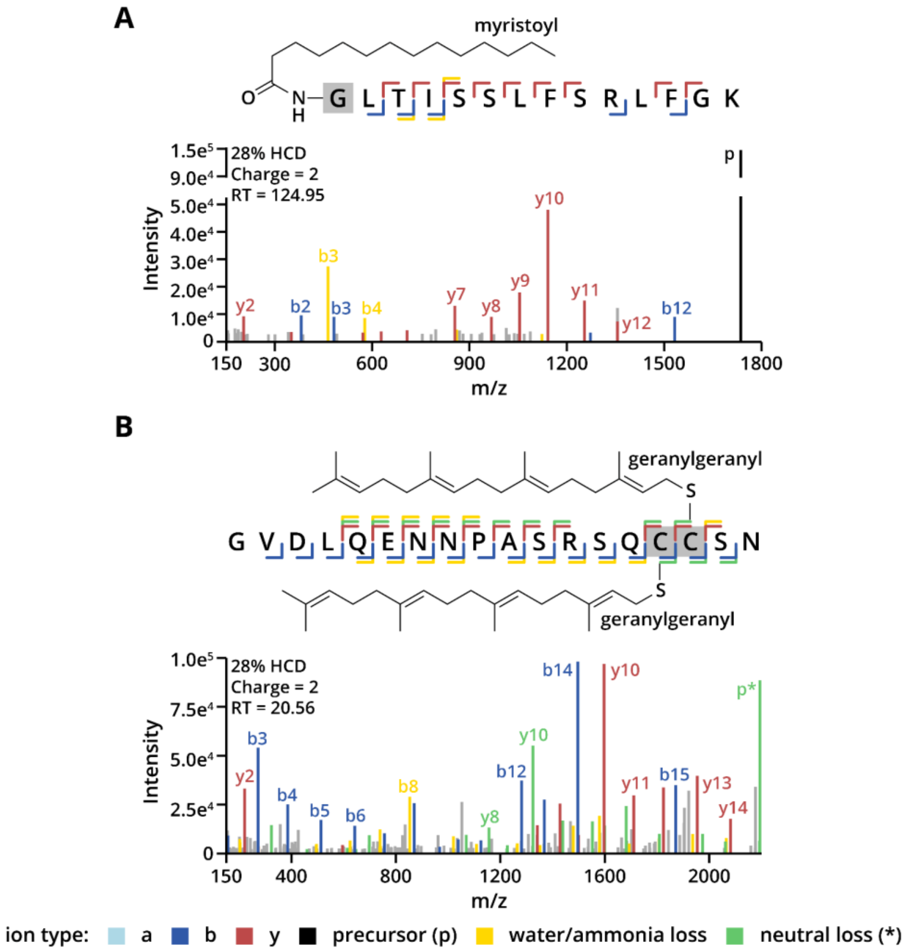

Supporting Figure 11. (A) MS/MS spectra of *N*-myristoylated ARF4 peptide fragmented with HCD. (B) MS/MS spectra of geranylgeranylated RAB5C peptide fragmented with HCD.
